# Autogenous dentin grafts in implant dentistry: A scoping review of clinical applications and processing protocols

**DOI:** 10.4317/medoral.27733

**Published:** 2025-10-17

**Authors:** Yuri Freires Braga, Eduardo Frederico Eduardo Maferano, Timóteo Sousa Lopes, Miguel Ribeiro-do-Nascimento Neto, Luis Eduardo Velez Macas, Fábio Wildson Gurgel Costa

**Affiliations:** 1 Oral and Maxillofacial Surgery and Traumatology at the Dr. José Frota Institute; 2 Department of Dentistry, Faculty of Health Sciences, Zambeze University, Tete City, Mozambique; 3 Postgraduate Program in Dentistry, Faculty of Dentistry, Federal University of Ceará, Ceará, Brazil; 4 School of Dentistry, Universidad Nacional de Loja, Ecuador

## Abstract

**Background:**

This scoping review aimed to systematically map and synthesize the current evidence regarding the use of autogenous dentin grafts (ADG) in bone augmentation procedures.

**Material and Methods:**

The review was conducted following the PRISMA-ScR (Preferred Reporting Items for Systematic Reviews and Meta-Analyses Extension for Scoping Reviews) guidelines. A comprehensive search was performed in MEDLINE (PubMed), EMBASE, CINAHL, Cochrane Library, Livivo, LILACS, Web of Science, Scopus, and ProQuest Dissertations &amp; Theses Global Google Scholar (gray literature) using the descriptors "Bone Regeneration," "Dentin," and "Dental Implants" combined with the Boolean operator and 7,690 studies identified through Google Scholar.

**Results:**

The initial search, conducted on December 23, 2024, retrieved 2,391 records, of which 16 met the eligibility criteria and were included in the final review. The findings indicate that autogenous dentin demonstrates efficacy in bone regeneration and alveolar ridge preservation, frequently exceeding the performance of allografts and xenografts. ADG was associated with increased bone density, enhanced implant stability, and improved soft tissue outcomes, particularly when combined with growth factors. Moreover, it was found to be a cost-effective alternative, producing outcomes comparable to other biomaterials regarding new bone formation and bone quality. Its osteoinductive properties further support long-term bone regeneration.

**Conclusions:**

Nonetheless, a lack of standardization in dentin processing protocols was noted across studies. ADG represents an effective and accessible option for implant-supported rehabilitation, and future research should focus on standardizing its clinical application.

## Introduction

Tooth extraction is one of the most commonly performed surgical procedures in dental practice, indicated for a range of clinical, orthodontic, prosthetic, or economic reasons, such as impacted teeth, extensive carious lesions, fractures, or unfavorable positioning (1). Despite its routine nature, tooth extraction initiates a cascade of complex physiological events, with alveolar bone remodeling representing a particularly critical process. Studies have demonstrated that within the first three months post-extraction, significant dimensional losses occur, with reductions of up to 50% in alveolar ridge height and width (2 , 3). These changes, primarily driven by bone resorption, present a substantial challenge for implant rehabilitation, potentially compromising both aesthetic outcomes and functional predictability.

To mitigate these deleterious effects, alveolar ridge preservation (ARP) strategies have been widely implemented, involving socket augmentation with biomaterials capable of maintaining bone volume and promoting tissue regeneration (2 - 5). Various approaches have been explored, including allografts, xenografts, synthetic materials, and, more recently, autogenous dentin grafts (ADG), an emerging alternative notable for its composition closely resembling bone tissue, as well as its osteoconductive and osteoinductive properties and high biocompatibility (6 - 8). Autogenous dentin, derived from the patient's own extracted teeth, offers significant regenerative potential. Its processing is relatively simple and rapid, and can be performed in a clinical setting without specialized laboratory facilities. This practicality contributes to reduced operative time, lower costs, and decreased morbidity associated with secondary donor sites-factors that enhance the procedure's acceptability among clinicians and patients alike (2 - 9).

Despite these advances, substantial gaps remain in understanding the mechanisms of action, clinical limitations, and standardization of dentin grafting techniques. In this context, the present scoping review aims to evaluate the effectiveness of autogenous dentin grafts as a bone substitute in implant dentistry, with a focus on processing methods, clinical limitations, and reported outcomes. Additionally, this review seeks to map the current state of evidence, identify knowledge gaps, and provide a comprehensive overview to inform evidence-based clinical decision-making and guide future research directions.

## Material and Methods

Scoping reviews are designed to comprehensively map and synthesize the available evidence on a specific topic and are particularly suitable for assessing the effectiveness of autogenous dentin grafts (ADG) in implant dentistry procedures (10). This scoping review was conducted in accordance with the PRISMA-ScR (Preferred Reporting Items for Systematic Reviews and Meta-Analyses Extension for Scoping Reviews) checklist (11) and followed the methodology recommended by the Joanna Briggs Institute (JBI) for scoping reviews (12). The protocol was prospectively registered on the Open Science Framework (https://osf.io/) under DOI: 10.17605/OSF.IO/9KTJC. The guiding research question was: "What is the effectiveness of autogenous dentin grafts as a bone substitute in implant dentistry, considering their processing methods and clinical limitations?"

Search Strategy

The search strategy was developed in three sequential stages to identify relevant studies across major databases.

First stage: A preliminary search was conducted in MEDLINE (via PubMed), EMBASE, CINAHL (EBSCO), The Cochrane Library, Livivo, LILACS (BVS), Web of Science, Scopus, and ProQuest Dissertations &amp; Theses Global Google Scholar (gray literature) to identify sentinel articles. Keywords were extracted from the titles and abstracts of these articles, in addition to controlled vocabulary terms (e.g., MeSH and Emtree), to construct a comprehensive search strategy. The descriptors employed included: "Bone Regeneration," "Dentin," and "Dental Implants," combined using the Boolean operator AND, while synonyms were combined using OR. The final search strategy was adapted to the syntax and indexing requirements of each database and is provided in Supplementary Material 1 (http://www.medicina.oral.com/carpeta/suppl1_27733).

Second stage: The main search was conducted on December 23, 2024, using the aforementioned databases.

Third stage: An updated search was performed on March 20, 2025, to identify additional studies that may have been indexed since the initial search.

Eligibility Criteria

This review included randomized clinical trials (RCTs) published in English that investigated the use of autogenous dentin grafts (ADG) as a bone substitute in procedures involving socket filling or coverage of bone sites. Eligible studies were required to assess the influence of ADG on bone regeneration and to be indexed in PubMed. The exclusive inclusion of RCTs was justified by their methodological rigor and ability to provide high-level clinical evidence, thereby minimizing bias and allowing a more accurate assessment of the effectiveness of ADG as a regenerative biomaterial.

Exclusion criteria included: RCTs conducted in patients with systemic conditions (e.g., syndromes affecting bone metabolism or metabolic bone diseases), studies involving pregnant or lactating women, case series, brief communications, letters to the editor, and pilot studies.

Study Screening and Selection

After identification, all records were exported to Rayyan software (http://rayyan.qrci.org, Qatar Foundation, Qatar), where duplicates were automatically removed. Screening was conducted in two stages:

First stage: Two independent reviewers (EFM and TSL) assessed titles and abstracts of all retrieved references. In cases of disagreement, a third reviewer (FWGC) with scoping review experience and subject expertise resolved discrepancies. Studies not meeting inclusion criteria were excluded.

Second stage: Full texts of selected articles were assessed against the same eligibility criteria to confirm inclusion. Evaluation was independently conducted by two reviewers (EFM and TSL), with final decisions validated by the third reviewer (FWGC). Final inclusion was based exclusively on full-text assessment, and reasons for exclusion were systematically documented.

Data Extraction

Data extraction was performed independently by two reviewers (YB and FWGC) using a predefined structured Microsoft Excel form to ensure consistency. Extracted variables included: Author, year of publication, study title, country of origin, sample size and number of groups, methodological design, main findings, techniques for processing autogenous dentin (including name, description, advantages, and disadvantages), methods for comparing ADG with other graft types, and study conclusions.

## Results

Search strategy and study selection

**Figure 1:**
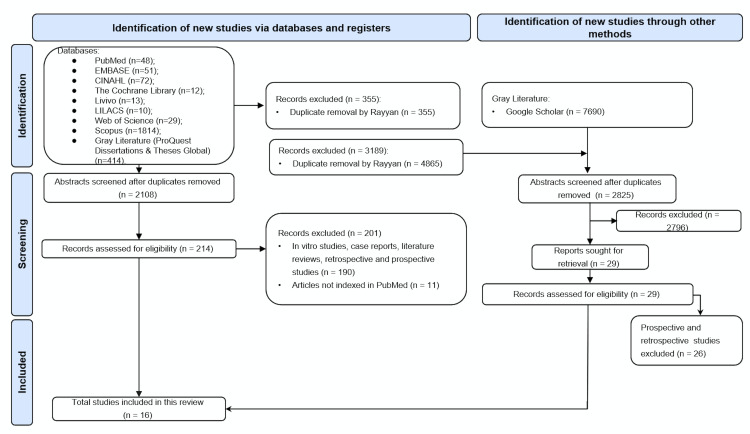
Flow diagram of literature search and selection criteria.

The systematic search across multiple databases yielded 2,463 records, with an additional 7,690 studies identified through gray literature searches via Google Scholar. After removing 355 duplicates using Rayyan software, 2,108 records remained for title and abstract screening. Of these, 214 articles were assessed in full text for eligibility. A total of 201 studies were excluded for failing to meet the inclusion criteria, primarily due to the absence of a randomized design (n=97), retrospective or uncontrolled prospective methodologies (n=51), classification as narrative reviews (n=42), or lack of PubMed indexing (n=11). Ultimately, 16 studies met all eligibility criteria and were included in the review. The detailed process of study identification, screening, eligibility assessment, and inclusion is illustrated in the PRISMA flow diagram (Figure 1) and Supplementary Material 2 (http://www.medicina.oral.com/carpeta/suppl2_27733).

1

Characteristics of the included studies

A total of 16 studies (2 , 3 , 4 , 5 , 6 , 7 , 13 , 14 , 15 , 16 , 17 , 18 , 19 , 20 , 21 , 22) were included, evaluating the performance of autogenous dentin grafts in terms of the quantity and quality of newly formed bone, implant stability in grafted sites, and overall procedural success. All selected studies adhered to ethical standards for human research and included randomized populations undergoing clinical grafting procedures with autogenous material derived from previously extracted teeth. Further details can be found in Supplement 3 (http://www.medicina.oral.com/carpeta/suppl3_27733).

**Figure 2:**
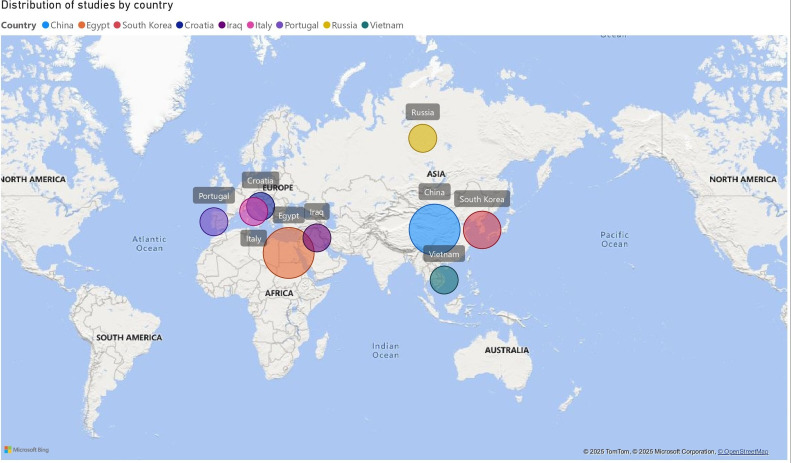
Distribution of studies by country.

Geographically, four studies (3 , 7 , 13 , 14) were conducted in Egypt, four (5 , 16 , 20 , 22) in China, and two (15 , 21) in South Korea. The remaining studies were conducted in Croatia (19), Iraq (6), Italy (17), Portugal (2), Russia (4), and Vietnam (18). The distribution of studies by country is illustrated in Figure 2, with further details in Supplement 3 (http://www.medicina.oral.com/carpeta/suppl3_27733).

2

The total sample size across studies was 540 patients, ranging from 11 to 80 participants per study (mean: 33.75 patients). Regarding comparisons, 11 studies (2 , 3 , 4 , 6 , 7 , 13 , 14 , 16 , 20 , 21 , 22) compared autogenous dentin grafts with either allografts or xenografts, representing the majority of included publications. The remaining studies compared autogenous dentin grafts with a combination of allografts and xenografts (15), a combination of xenograft and autogenous bone graft (19), or no grafting material (5 , 17 , 18). Three studies defined the control group as patients undergoing spontaneous alveolar bone healing without intervention (5 , 6 , 18 , 22).

Overview of clinical studies employing different forms of autogenous dentin grafts

The included studies investigated the clinical efficacy of various autogenous dentin graft forms for diverse indications in implant dentistry and bone reconstruction, addressing different types of alveolar defects and comparing their performance with alternative grafting materials.

For Autogenous Demineralized Dentin Matrix (DDM), three studies (5 , 16 , 18) reported favorable outcomes in alveolar ridge preservation and bone regeneration, demonstrating performance comparable to or exceeding that of collagen grafts or Bio-Oss®.

Regarding Autogenous Partially Demineralized Dentin Matrix (APDDM), two studies (20 , 22) showed positive results in bone regeneration and site preservation, with outcomes comparable to Bio-Oss® grafts or spontaneous healing.

The application of autogenous dentin blocks was examined in one study (15), demonstrating effectiveness similar to autogenous bone, with satisfactory integration and bone support.

In studies assessing ground dentin, five investigations (2 , 4 , 6 , 17 , 21) reported consistent outcomes in alveolar ridge preservation and bone regeneration, both in comparison with xenografts and across different surgical closure techniques.

Modifications in graft composition were explored in three studies (3 , 13 , 19), including combinations with i-PRF, variations in demineralization time, and mixtures with xenografts, suggesting that specific adjustments in graft preparation may enhance bone regeneration outcomes.

Finally, one study (7) evaluated untreated mineralized dentin grafts (UMDG), reporting satisfactory results comparable to xenograft controls despite the absence of chemical processing.

Type of Teeth Used

Third molars were utilized in four studies (14 , 15 , 18 , 22), demonstrating good bone integration and favorable healing responses. Premolars extracted for orthodontic purposes were employed in three studies (3 , 5 , 17), yielding satisfactory outcomes in maintaining bone volume. Anterior teeth and other non-molar maxillary teeth were used in three studies (6 , 13 , 19), particularly in esthetic zones, achieving adequate bone regeneration.

-Biological properties of dentin grafts

Autogenous dentin grafts demonstrated both osteoinductive and osteoconductive properties, providing structural support for bone formation and promoting new bone development (4 , 6 , 7 , 15 , 17 , 19 , 20 , 22). Demineralization of dentin enhanced the release of bioactive proteins from the collagen matrix, thereby increasing its regenerative potential (3 , 17).

Clinically and radiographically, the use of autogenous dentin in alveolar ridge preservation and bone augmentation significantly mitigated post-extraction ridge width and height loss (3 , 4 , 6 , 13 , 17 , 19 , 20 , 22). In one study, the mean reduction in buccolingual ridge width was 1.02±0.45mm with Autogenous Demineralized Dentin Graft (ADDG), which was lower than that observed in spontaneously healed sockets (3).

Additional benefits included preservation of keratinized tissue and reduced postoperative pain. Combining ADDG with injectable platelet-rich fibrin (i-PRF) resulted in less resorption of the keratinized mucosa (0.12±0.34mm vs. 0.58±0.34mm with ADDG alone) and lower immediate postoperative pain scores (13). The use of Autogenous Partially Demineralized Dentin Matrix (APDDM) in orthodontic patients also reduced perceived pain compared with bovine grafts (20).

Implant stability at sites grafted with Mineralized Dentin Matrix (MDM) was comparable to areas treated with xenogeneic biomaterials for both primary and secondary stability assessments (2 , 4 , 7 , 16 , 21).

Histological and histomorphometric analyses confirmed the regenerative potential of dentin grafts, with new bone formation ranging from 31.24% to 48.4%, depending on graft type and processing. ADDG achieved 48.4% new bone formation (3), dentin blocks 42.6% (14), and the AutoBT matrix 31.24%, a value comparable to Bio-Oss® (35%) (21). In some cases, dentin grafts outperformed xenogeneic materials; for instance, MDM yielded 47.3% new bone formation compared with 34.9% in the control group (2).

Graft integration was effective, with active remodeling and no significant inflammation observed (3 , 6 , 14 , 19). Demineralized dentin demonstrated superior tissue remodeling and integration compared with mineralized dentin, suggesting that demineralization enhances osteoinductive properties (3).

Processing protocols of autogenous dentin grafts

Protocols for processing teeth as autogenous grafts varied considerably across studies.

Demineralized dentin grafts (ADDG/DDM/APDDM) were most frequently described. Initial cleaning involved removal of restorations, carious tissue, enamel, pulp, periodontal ligament, and soft tissues using diamond burs, curettes, and specialized instruments (2 , 4 , 6 , 15 , 18 , 19 , 20). In one protocol, teeth were immersed in 3% hydrogen peroxide for 1-2 minutes to facilitate soft tissue removal (4).

Teeth were fragmented after drying using devices such as Smart Dentin Grinder® (2 , 4 , 6 , 18 , 19), BonMaker® Auto-Tooth Bone Graft (21), or AutoBT (15). Particle sizes ranged from 250-1200 µm, with particles smaller than 250-300 µm discarded (2 , 4 , 6 , 19 , 20).

Chemical protocols varied in agents and exposure times, including 0.6 N HCl for 30 minutes (3) or 17% EDTA for 2 minutes (18), while some studies did not specify the agent or duration (20).

For cleaning and disinfection, solutions included 0.5 M NaOH with 30% alcohol (2 , 4 , 6 , 18 , 19) or combinations of superoxide, ethanol, and distilled water (15). Particles were neutralized with phosphate-buffered saline (PBS) washes for 5 minutes (2 , 4 , 6 , 18 , 19).

Drying was performed using sterile gauze or air jet. The final material could be used immediately or stored under specific conditions, including freezing for dentin blocks (2 , 6 , 15).

**Figure 3:**
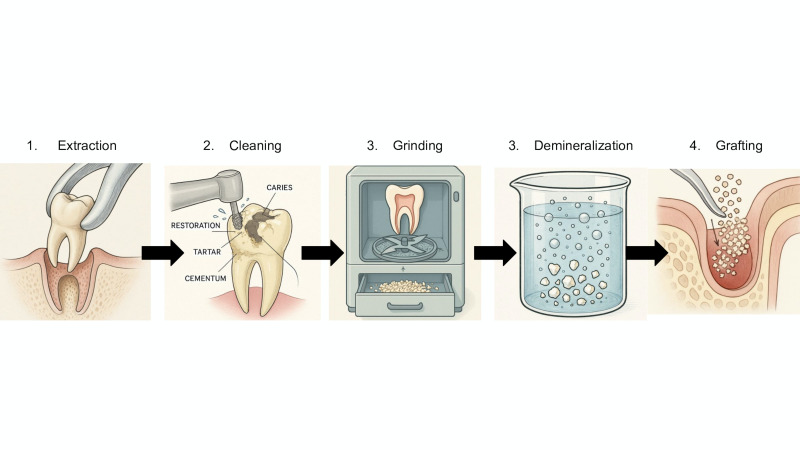
Schematic representation of the autogenous dentin graft processing protocol.

Untreated mineralized dentin grafts (UMDGs) followed a simpler protocol, limited to pulp removal and grinding, followed by rinsing with 0.9% saline solution, without further chemical treatment (7). The autogenous dentin graft processing workflow is illustrated in Figure 3.

3

## Discussion

This scoping review assessed the efficacy of autogenous dentin grafts (ADG) as a bone substitute in implant dentistry, focusing on processing protocols, clinical limitations, and reported outcomes. It also aimed to map the available evidence, identify knowledge gaps, and provide a comprehensive overview of the field. Most included studies were published within the past two years, reflecting growing interest in the topic and aligning with clinical and market demand for safe, effective, and cost-efficient solutions that expand treatment options and optimize workflow.

Geographic analysis revealed a predominance of studies conducted in South Korea, China, and Egypt, accounting for approximately 65% of the included articles (3 , 5 , 7 , 13 , 14 , 15 , 16 , 20 , 21 , 22). While these countries have substantially contributed to the literature, this concentration limits the generalizability of the findings, as the studied populations may not reflect the genetic, biological, and environmental diversity of other contexts. Moreover, the exclusive inclusion of PubMed-indexed randomized clinical trials (RCTs) may have excluded potentially informative studies from other databases or case series, which could have complemented the evidence by providing preliminary clinical insights or longer-term observational data. Future research should involve more diverse populations and consider broader sources of evidence to generate globally applicable findings, particularly in contexts where genetic factors influence treatment response and biomaterial metabolism (23 , 24).

ADG demonstrated clinical efficacy in immediate implant placement, reducing alveolar ridge resorption and providing high biocompatibility with minimal risk of immune rejection, particularly in patients with systemic or cultural restrictions (2 , 15 , 21). Optimal graft preparation-including removal of cementum, enamel, carious tissue, pulp, and soft tissues, followed by meticulous grinding, disinfection, and demineralization-is essential to ensure osteointegration and minimize complications (2 , 6 , 14). Devices such as the Smart Dentin Grinder® and BonMaker® facilitate standardization and reproducibility of clinical outcomes.

Studies reported clinical equivalence between ADG, PRP, Bio-Oss®, and AutoBT, suggesting that material selection may be influenced by cost, availability, patient preference, and clinician experience (15 , 21). Personalized material selection should also consider initial bone density, anticipated rehabilitation time, and individual patient characteristics.

Demineralized dentin grafts (DDG) showed benefits in secondary stability, whereas block grafts demonstrated increased bone volume and reduced resorption, offering an alternative to particulate grafts (14). However, the lack of direct comparisons between particulate and block grafts represents a significant research gap. Chemical disinfection and demineralization protocols using ethanol or acids expose bioactive growth factors, thereby enhancing bone regeneration (19), but require careful control to preserve essential proteins.

Beyond clinical efficacy, ADG offers economic advantages by reducing reliance on allografts and xenografts, thereby enhancing treatment accessibility and sustainability (6). Modern processing devices enable same-day graft preparation, reducing operational costs and improving clinical autonomy. Although the initial investment is substantial, per-patient costs decrease over time while workflow efficiency and patient access improve.

This review has several limitations. First, substantial heterogeneity in dentin processing protocols (such as differences in enamel and cementum removal, grinding techniques, demineralization agents, and disinfection methods), hampers direct comparison across studies and limits the development of standardized clinical guidelines. Second, the geographic concentration of available studies, combined with the restriction to PubMed-indexed randomized clinical trials, further constrains the generalizability of the findings. While case series or studies retrieved from additional databases could have provided complementary evidence-particularly regarding rare complications, longer-term outcomes, or preliminary efficacy in diverse patient populations-we deliberately limited eligibility to RCTs in order to ensure a higher level of methodological rigor. These factors should be taken into account when interpreting the conclusions of the present review.

Despite these limitations, this study has notable strengths. This scoping review offers a comprehensive synthesis of clinical applications, processing protocols, and outcomes of ADG in implant dentistry. To our knowledge, it represents the first review to perform a comparative evaluation between ADG and other graft materials, highlighting its efficacy, versatility, and potential advantages for clinical practice. These findings provide a robust foundation for future research and support evidence-based therapeutic decision-making.

## Conclusions

Autogenous dentin grafts (ADG) represent a promising bone substitute, particularly for patients with systemic or cultural limitations. Their clinical efficacy depends on meticulous preparation, including thorough cleaning and demineralization. However, the lack of standardized processing protocols and procedural variability limits cross-study comparability and constrains the generalizability of current evidence. While ADG reduces dependence on allogeneic and xenogeneic materials-enhancing cost-effectiveness and sustainability-it also presents challenges, such as the need for long-term safety evaluation, potential risks of cystic or neoplastic cell contamination, and inconsistencies in graft processing. Future investigations should prioritize protocol standardization, inclusion of more diverse populations, and resolution of methodological gaps to ensure safety, reproducibility, and broad clinical applicability in implant dentistry. Specifically, multicenter randomized trials employing standardized dentin processing protocols, along with comprehensive evidence syntheses incorporating diverse study designs and databases, are essential to strengthen generalizability and clinical relevance.

## Data Availability

Declared none.
